# Editorial: Plant and human pathogen interactions: gaining insights into the impact of environmental and crop management factors, volume II

**DOI:** 10.3389/fpls.2026.2008165

**Published:** 2026-10-09

**Authors:** Cristian Jacob, Massimiliano Marvasi, Shirley A. Micallef

**Affiliations:** 1 Departamento de Ciencias Vegetales, Facultad de Agronomía y Sistemas Naturales, Pontificia Universidad Católica de Chile, Santiago, Chile; 2 Department of Biology, Università degli Studi di Firenze, Sesto Fiorentino, Florence, Italy; 3 Department of Plant Science and Landscape Architecture, University of Maryland, College Park, MD, United States; 4 Centre for Food Safety and Security Systems, University of Maryland, College Park, MD, United States

**Keywords:** antimicrobial resistance, food safety, fresh produce, human norovirus, *Salmonella enterica*, surface topography, topomimetics, urban agriculture

Promoting increased consumption of fresh fruits and vegetables remains a cornerstone of global public health strategies aimed at preventing noncommunicable diseases and addressing micronutrient deficiencies. However, the safety of fresh produce continues to be challenged by contamination with human enteric pathogens. The persistence and dissemination of human pathogens within agricultural ecosystems represent a complex interplay between microbial physiology, plant-associated niches, environmental stressors, and crop management practices. Following the insights gathered in Volume I of this Research Topic, Volume II brings together four contributions that advance our understanding of these dynamics. Together, these studies reveal how biological adaptation, produce surface characteristics, and environmental reservoirs influence the persistence, transmission, and control of human pathogens and antimicrobial resistance within agricultural systems.

A foundational element of pathogen persistence is the ability of human enteric bacteria to adapt to non-host, plant-associated environments. In their comprehensive review, Han et al. synthesize the metabolic adaptations utilized by *Salmonella enterica* to survive and colonize plant tissues and agricultural substrates. The authors delineate how *S. enterica* dynamically reprograms its central carbon and nitrogen metabolism in response to nutrients available in root exudates, leaf apoplastic fluids, and plant tissues. Specifically, *S*. *enterica* prioritizes glucose metabolism through glycolysis, shifts to gluconeogenesis under sugar-limiting conditions, and exploits alternative carbon sources such as glycerol, fatty acids, and organic acids. Furthermore, *de novo* amino acid biosynthesis, particularly cysteine via sulfate assimilation, emerges as an indispensable metabolic hub required for bacterial fitness across diverse plant hosts and stress conditions. By highlighting this metabolic plasticity, Han et al. provide insights into how human pathogens exploit plant metabolic niches, identifying physiological vulnerabilities that could be targeted to prevent pre-harvest colonization.

Interactions at the plant surface are relevant for pathogen attachment, retention, and susceptibility to post-harvest sanitation. Addressing this physical interface, Lightbown and DiCaprio investigated the impact of leafy green microscale surface topography on the recovery and chemical sanitizer inactivation of Tulane virus (TV), a surrogate for human norovirus. Comparing fresh romaine lettuce and spinach leaves across growth stages and leaf axes, the authors demonstrated that spinach leaf maturity significantly influences chemical inactivation: younger leaves allowed higher viral inactivation by sodium hypochlorite than older leaves. Crucially, the authors engineered polydimethylsiloxane (PDMS) topomimetic artificial leaf surfaces (“replicasts”) to decouple physical structural attributes from biological variability. By modulating replicast hydrophobicity with low-concentration surfactants, the artificial surfaces faithfully reproduced viral recovery trends observed on fresh plant tissue, validating topomimetic replicasts as standardized physical tools for produce safety research.

Expanding this physical engineering framework to soft fruits, Lightbown and DiCaprio evaluated PDMS topomimetic artificial surfaces modeled after blueberries, strawberries, and raspberries. Soft berries present intricate surface features, ranging from smooth epicuticular wax on blueberries to achene pits on strawberries and dense trichomes on raspberry drupelets, that create micro-refuges protecting viruses from post-harvest wash treatments. Their findings demonstrated that berry type markedly dictates viral retention, with raspberries retaining higher viral titers than blueberries or strawberries. Surface replicasts of all three berry types closely mirrored the recovery levels obtained from fresh fruits, confirming that physical micro-architecture is a primary driver of viral adhesion and protection against sanitizer wash solutions. These data indicate that while berry replicasts may have utility in modeling surface adhesion of viruses to berries and in viral recovery studies, they are not suitable for studies of viral inactivation by chemical sanitizers.

Beyond pathogen persistence and sanitizer efficacy, agricultural environments are increasingly recognized as reservoirs of antimicrobial resistance (AMR), introducing an additional dimension to produce safety risk assessment. Harrelson et al. conducted whole-genome sequencing on 87 multidrug-resistant (MDR) bacterial strains isolated from leafy greens, root zone soil, and bulk soil within urban agriculture systems. Representing 14 genera dominated by *Pseudomonas*, *Serratia*, *Providencia*, and *Bacillus*, these environmental isolates exhibited extensive resistomes primarily encoding multidrug efflux systems, β-lactamases, and tetracycline resistance determinants. All MDR isolates harbored mobile genetic elements (MGEs), with multiple antibiotic resistance genes (ARGs) located in close physical proximity (<5 kb) to transposons, plasmids, or prophages. These findings support the notion that urban agricultural soils and fresh produce can act as environmental reservoirs and potential mobilization vectors for AMR elements, emphasizing the necessity of monitoring resistome dynamics alongside conventional foodborne pathogens.

Overall, these studies demonstrate that both biological adaptation and environmental context shape the persistence, transmission, and control of food safety hazards in agricultural systems. Importantly, these findings also underscore how crop-management decisions may influence food-safety risks across production systems. Deciphering bacterial metabolic flexibility, standardizing physical produce surface models for viral wash treatments, and mapping environmental resistomes in urban agroecosystems collectively inform risk assessment, agronomic policy, and post-harvest intervention protocols. Han et al. highlight the remarkable metabolic adaptability of *S*. *enterica* to plant-associated environments, suggesting that differences in plant genotypes and their associated nutrient profiles may influence pathogen colonization potential. Lightbown and DiCaprio, Lightbown and DiCaprio demonstrate that harvest maturity, produce type, and post-harvest washing practices affect viral retention and sanitizer efficacy through differences in surface architecture. Likewise, Harrelson et al. show that urban agricultural soils and produce can serve as reservoirs of antimicrobial resistance, emphasizing the importance of understanding how management practices, including irrigation and soil amendment strategies, influence the composition and dissemination of resistance determinants. Together, these management factors interact with environmental conditions, including moisture, temperature, soil properties, and plant developmental stage, to shape pathogen persistence and transmission within agricultural ecosystems. This improved understanding of how enteric pathogens interact with fruit and vegetable production systems moves the field beyond solely exclusionary approaches to food safety and supports the development of more targeted interventions that address the biological and ecological mechanisms underlying pathogen survival. Continued research at the intersection of plant biology, microbiology, and agricultural systems remains essential to advance the safe and sustainable production of fresh fruits and vegetables globally.

